# Schwannoma of the breast presenting as a painful lump: A case report

**DOI:** 10.1097/MD.0000000000027903

**Published:** 2021-12-10

**Authors:** Han Shin Lee, Eun Jung Jung, Jae Myung Kim, Ju Yeon Kim, In Kyeong Kim, Jae Ri Kim, Tae Han Kim, Jae Yool Jang, Jung Woo Woo, JinKwon Lee, Taejin Park, Sang Ho Jeong, Eun Cho, Dae Hyun Song

**Affiliations:** a Department of Surgery, Gyeongsang National University College of Medicine and Gyeongsang National University Changwon Hospital, Changwon, Korea; b Department of Surgery, Gyeongsang National University College of Medicine and Gyeongsang National University Hospital, Jinju, Korea; c Department of Radiology, Gyeongsang National University College of Medicine and Gyeongsang National University Changwon Hospital, Changwon, Korea; d Department of Pathology, Gyeongsang National University College of Medicine and Gyeongsang National University Changwon Hospital, Changwon, Korea.

**Keywords:** breast, painful mass, schwannoma

## Abstract

**Rationale:**

Schwannoma in the breast parenchyma is very unusual. It usually develops on the head, neck, and extensor surfaces of the upper and lower extremities.

**Patient concerns:**

We report a case of a 60-year-old woman with a palpable and painful mass. Clinically, she experienced neuropathic pain at the mass site.

**Diagnoses:**

The tumor was a 1 cm, well-circumscribed mass, and revealed schwannoma on core needle biopsy

**Interventions:**

The patient underwent wide excision.

**Outcomes:**

No postoperative complications were observed. A six-month follow-up revealed no recurrence

**Lessons subsections:**

Although breast schwannoma is a very rare tumor, it is a very important consideration in case of a Breast Imaging-Report and Data System 4A lesion with a painful and palpable mass.

## 1 Introduction

Schwannomas are usually benign, slow-growing, and relatively rare tumors that develop on the head, neck, and extensor surfaces of the upper and lower extremities.^[1]^ They can arise from Schwann cells of any nerve in any organ.^[1]^ However, intramammary schwannomas are extremely rare, accounting for only 2.6% of all schwannomas.^[2]^ A review of schwannoma cases in the literature is limited.^[2]^ Herein, we present a case of a 60-year-old woman with a palpable and painful schwannoma in the breast.

## 2 Case report

### 2.1 Clinical summary

**Figure 1:**
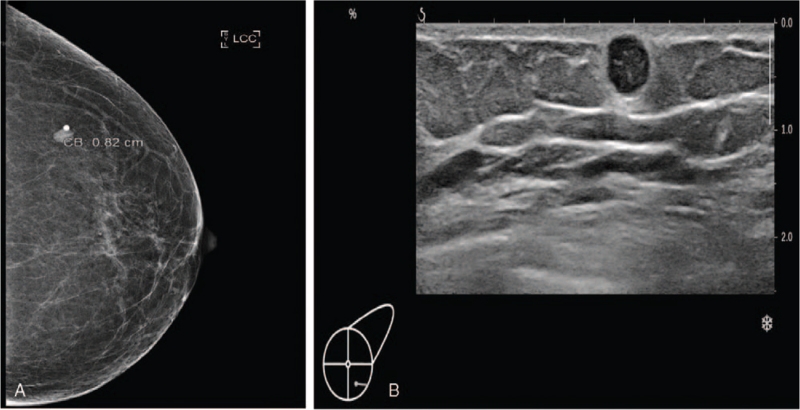
(A) Mammography shows a 0.8 cm circumscribed oval hyper-dense nodule at left breast. (B) Sonography shows a 0.6 cm round hypo-echoic nodule with posterior acoustic shadow at left breast.

**Figure 2:**
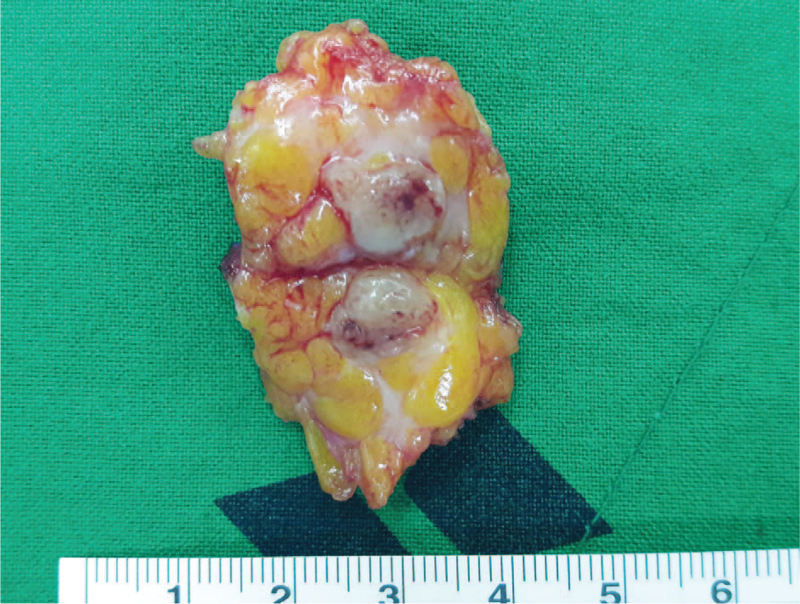
Gross specimen of left breast schwannoma after surgical excision.

A 60-year-old woman with no relevant medical history and no von Recklinghausen disease visited our department because of left breast pain. The pain site was the left lower outer quadrant breast, and a palpable, movable mass was observed. When the mass was touched, she felt weak nervous pain like an electronic shock at the left shoulder area and at that site. There were no palpable axillary lymph nodes in either axillae. Mammography showed a circumscribed, oval, and hyper-dense nodule at the left 4 o’clock position (Fig. 1A). Sonography revealed a 6-mm round, hypoechoic nodule with internal vascularity, for which ultrasound-guided core needle biopsy was recommended (Fig. 1B). As the lesion fell under the category of Breast Imaging-Report and Data System (BI-RADS) 4A, it was suspected to be malignant. An ultrasound-guided core biopsy was performed, and the specimen revealed a schwannoma. The patient underwent excisional biopsy, and the well-encapsulated mass was completely excised (Fig. 2). As of the patient's latest clinic visit 1 year after surgery, she had no pain or complications.

### 2.2 Methods

As this case report does not involve a prospective or retrospective study, the consent of the patient was sufficient, and ethical approval was not required. Thus, we decided to publish only the age, image findings, and pathological images in the case report, and we have received written consent from the patient for the same.

### 2.3 Pathological findings

**Figure 3:**
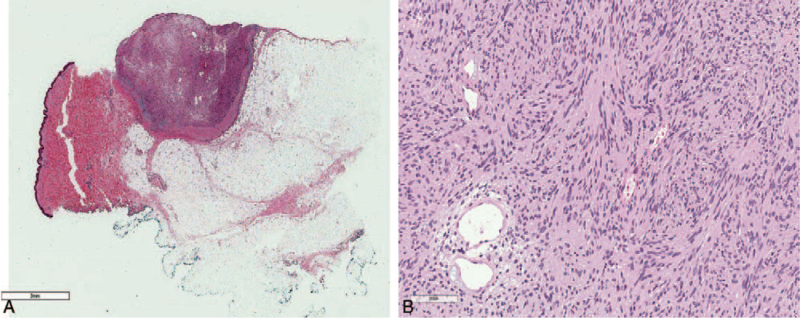
(A) There is a well-circumscribed nodule in subcutaneous area (H&E ×1). (B) The nodule is composed of mainly benign spindle cells and scattered dilated medium sized vessels. There are several areas with vague palisading pattern that looks like a Verocay body of spindle tumor cells (H&E ×200).

**Figure 4:**
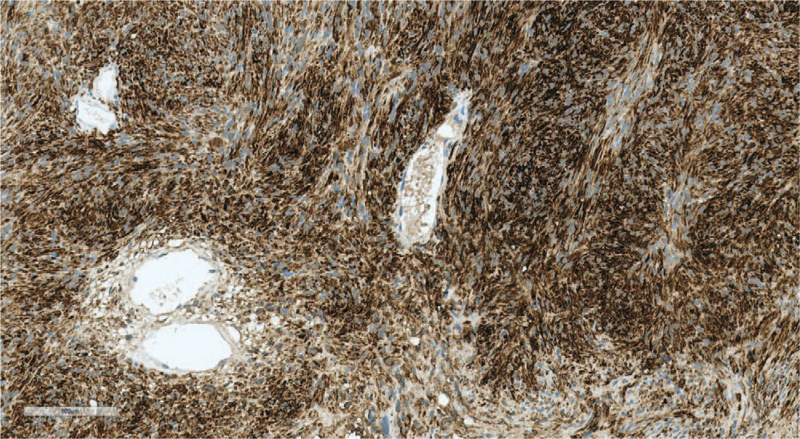
The spindle tumor cells show strong reactivity with the S100 immunohistochemical stain (S-100 ×200).

The tumor size was 1.0 cm, with a well-circumscribed negative margin (Fig. 3A). The final pathologic diagnosis was schwannoma (Fig. 3B). Immunohistochemical staining of the schwannoma revealed the presence of the following: Calponin-1 (+), epidermal growth factor receptor (EGFR) (–), tumor protein 63 (P63) (–), and S-100 (+) (Fig. 4).

## 3 Discussion

Schwannomas are derived from Schwann cells that form the myelin sheath of nerves, which facilitates the transmission of an impulse.^[3]^ A schwannoma is a slow-growing tumor that develops in the peripheral nerves or spinal roots.^[3]^ Any part of the body can be affected, but intramammary localization is rare. Breast schwannomas account for only 2.6% of all schwannomas, with an incidence of 0.2% of all breast cancer cases.^[1]^ A review of the English literature shows that there are only few reported cases.^[1]^ Only 3 cases have been reported in Korea.^[4]^ In all of these cases, for most tumors, the size ranged from 7 to 11 cm and they were located in the upper outer quadrant.^[4]^ In the present case, the tumor size was 1 cm, and the tumor was located in the left lower outer quadrant.

Pathologically, schwannomas have 2 components, known as Antoni A tissue and B tissue, in variable proportions, and these spindle cells show nuclear palisading and parallel arrays are known as Verocay bodies.^[3]^ In addition, schwannomas express S-100 protein on immunochemistry findings. Similarly, on cytological diagnosis of the present schwannoma case, the spindle cell was a vague palisading pattern that resembled a Verocay body and S-100 was positive. However, the Antoni A and Antoni B areas were not definite. The breast is mostly supplied by 2 nerves, the anterior and lateral cutaneous branches of the thoracic intercostal nerves, while also being innervated by the cervical plexus. Breast schwannoma is a peripheral nerve sheath tumor (PNST) in these nerves. Ogose et al^[5]^ reported that only 5/99 (5%) patients with benign schwannomas experienced pain at rest. In contrast, 94/99 (95%) patients with benign PNST had pain induced by pressure. In the present case, the patient experienced pain during mechanical stimulation of the mass. In other case reports, it can be very difficult to distinguish a schwannoma from other benign or malignant tumors on imaging findings.^[4]^

Clinically, a breast schwannoma is considered a BI-RADS 4A lesion that has a very low risk of malignant transformation.^[6]^ Therefore, core-needle biopsy should be performed for pathological diagnosis and for the purpose of treatment because of the recurrent nature of the breast tumor. Surgical excision is the treatment of choice for breast schwannomas and has a good prognosis.^[7,8]^ Therefore, benign schwannoma should be considered when a breast mass is palpable and painful with BI-RADS 4A lesions. The mass should be subjected to core-needle biopsy or excisional biopsy.

In conclusion, we report a case of breast schwannoma, which is a rare tumor and its characteristics. This neoplasm is very difficult to differentiate due to its morphological features and location. It is important to recognize this tumor based on physical examination and imaging findings. Above all, pathologic findings should confirm the type of tumor.

## Author contributions

**Conceptualization:** Han Shin Lee, Eun Jung Jung

**Data curation:** Han Shin Lee

**Formal analysis:** Han Shin Lee

**Investigation:** Han Shin Lee, Eun Cho, Dae Hyun Song

**Methodology:** Ju Yeon Kim

**Project administration:** Jae Myung Kim

**Resources:** Han Shin Lee, Eun Cho, Dae Hyun Song

**Supervision:** Eun Jung Jung

**Validation:** Jae Ri Kim, Tae Han Kim, Jae Yool Jang, Jung Woo Woo, JinKwon Lee, Taejin Park, Sang Ho Jeong

**Writing – original draft:** Han Shin Lee, Eun Jung Jung

**Writing – review & editing:** Eun Jung Jung
